# Discontinuance of the Dental Miscellany

**Published:** 1882-02

**Authors:** 


*Dental Miscellany—Discontinuance.*—We are sincerely sorry to see the discontinuance, with the December (1881) number, of Johnston's *Dental Miscellany.* It has been so sprightly, clever, and withal sensible, that its monthly visitation was always looked forward to with pleasure, and it was read with profit. We are sorry it goes out of existence. H.

